# Don't be a nocebo! Why healthcare organizations should value patients' expectations

**DOI:** 10.3389/fpsyg.2024.1393179

**Published:** 2024-04-17

**Authors:** David Poulter, Maxi Miciak, Jerry Durham, Alvisa Palese, Giacomo Rossettini

**Affiliations:** ^1^MT3 Clinical Education and Consulting, Coon Rapids, MN, United States; ^2^Faculty of Rehabilitation Medicine, University of Alberta, Edmonton, AB, Canada; ^3^Client Experience Company, Los Angeles, CA, United States; ^4^Department of Medical Sciences, University of Udine, Udine, Italy; ^5^School of Physiotherapy, University of Verona, Verona, Italy; ^6^Department of Human Neurosciences, University of Rome “Sapienza Roma”, Rome, Italy; ^7^Musculoskeletal Pain and Motor Control Research Group, Faculty of Sport Sciences, Universidad Europea de Madrid, Madrid, Spain; ^8^Musculoskeletal Pain and Motor Control Research Group, Faculty of Health Sciences, Universidad Europea de Canarias, Tenerife, Spain

**Keywords:** nocebo effects, expectation, placebo effects, patient, care

## 1 Introduction

Healthcare organizations, whether public or private, must continually address operational challenges that threaten their survival. In addition to staff recruitment and development, cost control, quality improvement, and technological innovation, healthcare organizations have recently become more interested in managing the expectations of their service users (i.e., the patients) (Crisafulli et al., 2019). Expectation represents a conscious phenomenon directed toward future events (Rief and Petrie, 2016). In healthcare, patient expectation is a multidimensional concept comprised of various elements that collectively contribute to its determination (Lakin and Kane, 2022). For instance, expectations can be considered in terms of what might happen (i.e., ‘probability expectation'), what will happen (i.e., ‘value expectation'), or the anticipated benefits of a treatment (i.e., treatment/care expectation) (Lakin and Kane, 2022). Moreover, an expectation is not predetermined (Sinatti et al., 2022) but changes based on the patient's direct experience with individuals within the organization, the information the patient is provided, and the experience of others observed by the patients (Rossettini et al., 2022).

The complexity underlying patient expectations suggests that healthcare organizations should consider them more broadly (Berhane and Enquselassie, 2016), with the intention of preventing possible negative nocebo effects (Rossettini et al., 2022). In the organizational context, nocebo effects are those negative effects triggered by any of its team members (e.g., leaders, managers, front-desk staff, clinicians) whenever they neglect, overlook, or do not meet patient expectations (Rossettini et al., 2020). When the organization does not adequately manage patient expectations, the subsequent nocebo effects could induce negative consequences both for patients and the organization (Villafañe et al., 2016; Rossettini et al., 2020). For example, an organization acting as a nocebo could create negative experiences along the patient's care pathway, possibly exacerbating their symptoms and worsening their clinical condition (Rossettini et al., 2023). Moreover, an organization not meeting patient expectation could result in dissatisfied patients, which in turn could have negative consequences on the patient's commitment to their treatment plan, as well as negative effects on word-of-mouth referrals and patient retention (Yetman et al., 2021; Connor et al., 2023). Finally, an organization with a high patient drop-out rate could lead to frustrated and demotivated team members, manifesting in increased absenteeism and employee turnover (Edwards-Maddox, 2023).

Although nocebo effects and expectations have been extensively investigated within experimental paradigms in the laboratory (Bagarić et al., 2022; Rooney et al., 2023) and care settings (Rooney et al., 2024), we believe there is a need to look beyond the clinician-patient interaction to embrace the complexity offered by healthcare organization at large. Accordingly, we propose that it is necessary to stimulate a debate on how different team members in a healthcare organization can inadvertently cause nocebo effects when patient expectations are either not considered or not met, along their care journey (Rossettini et al., 2020). To bridge this gap, in this opinion paper, we focus on how the organization as a collective of individuals enacting policies, practices, and processes can contribute to nocebo effects due to a disconnect in managing and meeting patients' expectations. Our main objectives are to: (1) describe potential scenarios of nocebo effects within the healthcare organization (i.e., not just individual professionals and/or the clinical interaction in isolation), and (2) suggest implications for their management.

## 2 Discussion

### 2.1 Scenarios of nocebo effects within the healthcare organization

**Figure 1:**
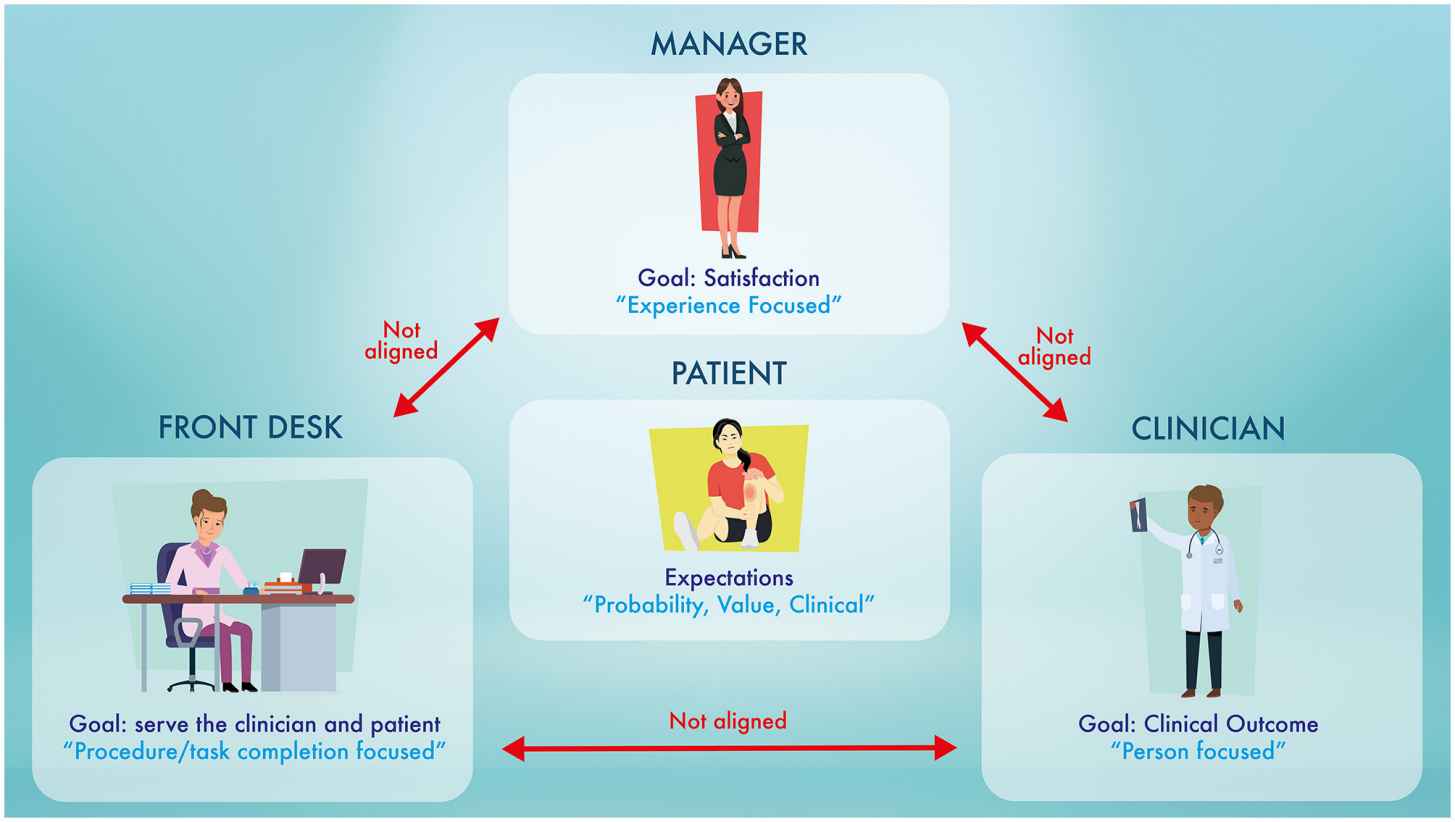
The “perfect storm.” This diagram highlights the different focuses of each of the members in the healthcare organization. This disconnect between the focuses of each player is the possible fuel that feeds the nocebo effects. Nocebo effects in a healthcare organization are “*silent like a cancer grows”* (homage to Simon and Garfunkel).

An organization must be clear about how patient expectations are addressed by its leaders, managers, clinicians, and support staff. We have proposed three scenarios, based on the authors' real-world experiences of healthcare organization practices, processes, and policies, that illustrate when patient expectations may not be given appropriate attention, potentially contributing to nocebo effects (Figure 1).

The first scenario illustrates how an organization can contribute to nocebo effects when there is a difference between what it says it is going to do and what it actually does, especially related to patient expectations for care. An organization communicates to patients what it is going to do through its mission statements, goals, and values, which creates expectations for their care. For instance, healthcare organizations may claim they value a “person-centered”, person-focused', or “collaborative”, approach to care (which also happens to be models that prioritize patient expectations). (Bellows et al., 2014). However, challenges can arise with implementing these person-focused care models because they may clash with other organizational priorities. For example, a clinician's ability to effectively provide person-focused care can be severely affected by an organization's decisions to reduce appointment duration and increase clinicians' caseload resulting in unmet patient expectations of care (O'Keeffe et al., 2016). A disconnect between “what is said” and ‘what is done may occur when managers become beholden to performance metrics that prioritize efficiency over appropriateness of care (Health Quality Council of Alberta, 2017). This disconnect may inadvertently reinforce and reward clinician attitudes and behaviors that are incongruent with what patients expect based on the organization's values.

The second scenario reveals how nocebo effects could manifest due to a disconnect with the level at which clinicians and managers view patient expectations (Mannion and Davies, 2018), negatively affecting organizational synergy. Clinicians view patients as individuals and determine patient expectations by using strategies that help identify person-focused needs and goals (e.g., shared decision-making) (Hutting et al., 2022). Consequently, they assess success by subjectively inquiring about patients experiences and objectively measuring clinical outcomes such as symptom reduction and functional improvements in order to make individualized clinical decisions thereby addressing patient expectations of clinical care. Conversely, managers tend to view patients as groups and focus on measuring patient experience (e.g., satisfaction) in aggregate in order to make operational decisions that address patient expectations on the whole (Mannion and Davies, 2018). Although reasonable considering their different roles, discord can develop between the clinicians and managers if organizations do not ensure everyone understands the value of addressing the scope of patient expectations, especially when satisfaction and clinical measures do not match (Garth et al., 2013). For example, patients may report high satisfaction with their care experience despite poor clinical outcomes (Prang et al., 2019). This could result in negative feelings, for example, if managers consciously or unconsciously place less emphasis on achieving clinical objectives (e.g., by deciding to reduce expenditures for appropriate rehabilitation equipment), thereby affecting patient expectations of quality and outcomes of clinical care.

The third scenario of nocebo effects comes from the role front-desk staff (e.g., receptionists) play in addressing patient expectations. Healthcare organizations are made up of multiple actors who all contribute to creating a positive context, with the good work of some undone by others (Martin and Waring, 2013). Front desk staff are frequently overlooked but play an important role in the patient's care journey because they are often the first point of contact in the organization. However, conflict is common when patients interact with receptionists, causing distress (Hewitt et al., 2009). The typical communication style among medical receptionists in the UK is task-centered, conventionally polite and rapport-building (Hewitt et al., 2009). However, some suggest that being too task-centered can lead to negative effects (nocebo effects) with patients who perceive receptionists as over-direct. Additionally, nocebo effects could occur when an organization's front-desk staff have not been adequately trained to consider patient expectations (Manning et al., 2012). For example, receptionists are often charged with multiple important tasks that many are not prepared for by the healthcare organization. It has been shown that many receptionists have to decide who to prioritize for appointments, often making unqualified triage decisions which can directly affect patient care and clinical outcomes (Litchfield et al., 2017).

A perfect storm of nocebo effects that exemplifies what we have presented above may be the UK National Health Service (NHS), as recently reported in the UK press (Ennals, 2023). To address long musculoskeletal waiting lists, NHS is under pressure to reduce wait times and find solutions for a GP shortage (Halls et al., 2020; Ennals, 2023). This pressure has contributed to ‘First Contact Physiotherapy', a model of care used in the UK NHS whereby specialist physiotherapists assess, diagnose, and manage patients traditionally first seen by GPs (Chartered Society of Physiotherapy, 2018; Halls et al., 2020). Purported benefits for patients include quicker access to expert musculoskeletal consultation, longer appointment times, and an improved sense of being heard and cared for (Chartered Society of Physiotherapy, 2018). However, there have been reports of reductions in appointment duration (e.g., from 20-minutes to 10-minutes), with practice managers sometimes pressuring physiotherapists to do so (Halls et al., 2020), possibly to reduce wait lists. This could leave physiotherapists dissatisfied with work and frustrated because they cannot practice in a person-focused manner. Some patients have complained that physiotherapy sessions are too short, that they have not been heard, and that they received generic advice and exercise sheets (Ennals, 2023). Further upstream, front-desk staff may be met with angry patients who expect to see their GP and feel they are being “fobbed off” to what they perceive, albeit inaccurately, as lesser qualified clinicians. This all amounts to the strong potential for nocebo effects due to a serious breach of patient expectations, perpetuated by unhappy front staff, overworked clinicians, and stressed managers.

### 2.2 Implications for the healthcare organizations

Organizations can induce nocebo effects when patient expectations are not addressed, as illustrated by the three real-world scenarios. While we cannot offer specific solutions given each organization operates within its own context, we offer considerations to increase awareness of what organizations could do to contain nocebo effects (Table 1).

**Table 1:** Key points.

**Scenarios of nocebo effects within a healthcare organization**
• The difference between what an organization says it is going to do and what it actually does related to how it operationalises patients' expectations. • Tension between the organization's management and clinicians based on their different views of what patients' expectations are and how they are assessed. • The possible disconnect and lack of training of front-desk staff within the organization, leading to improper management of patients' expectations.
**Implications for the management of nocebo effects in healthcare organizations**
• Engaging patients in meaningful roles on boards, committees, or working groups to help integrate patient expectations throughout organizational decision-making. • Targeting health care practitioners for managerial or other leadership positions. • Training managers, clinical staff, and ancillary support staff in one chosen person-focused approach. • Providing organization-wide training to facilitate effective communication, top down and bottom up. • Aligning all parts of the organization's outcome measures so they reflect patient values and expectations. • Training in conflict management to prevent nocebo effects due to tensions between different actors throughout the organization. • Ensuring front-desk staff are operating within the scope of their role and responsibilities to prevent negative effects from conflicting expectations of care.

We believe organizations can take action to prevent nocebo effects by better aligning their objectives with how they operationalize care — specifically, by weaving patient expectations into their governance and quality evaluation processes. Patient engagement in healthcare decision-making is a growing phenomenon in Western countries (Fancott et al., 2018). Genuinely involving patients on boards, committees, and working groups is one way to integrate valuable contributions that could transform how patient expectations inform organizational policies and practice. As Peter Drucker said, “what gets measured will likely get done”. Therefore, specific performance indicators/metrics related to patient expectations can reinforce person-focused decisions and actions (Starfield, 2011) by leaders, managers, clinicians, and support staff. Further, organizations are more likely to develop care pathways and provide necessary resources if they are being held accountable by performance measures.

We have proposed that a difference and lack of understanding between how clinicians and managers view patient expectations could result in nocebo effects. We suggest organizations adopt ways to consolidate information about how and why patient expectations are being addressed to enhance mutual understanding and communication amongst organizational actors (Chandrashekar and Jain, 2019). It may also help to have individuals with clinical experience in managerial positions. Research suggests that organizations that hire clinicians in managerial roles have better clinical outcomes and overall satisfaction compared to organizations without (Lega et al., 2013). This may be due to the clinicians' ability to empathize with the challenges faced in the clinical trenches.

Organizations must create the conditions for successfully managing and setting patient expectations, from first contact to discharge and beyond. This involves mapping out, understanding, and training all team members in the organization about the patient's entire journey (Clear Survey, 2024). Staff training and effective communication should be emphasized with a clear mission of placing the patient and their expectations as the main focus of the whole organization. Viewing patients through this person-focused lens throughout the organization may lead to more positive outcomes and satisfaction and decrease the amount of nocebo effects occurring within the organization (Garth et al., 2013). This will help managers, clinicians, and front-desk staff to synergize with one another and the patient's expectations as well as enhance their capacity to take ownership over their particular roles in creating a context for patient and organizational success. In recognition that front-desk staff are the first contact for most patients accessing a healthcare organization, it is very important to ensure they are well-trained and not expected to make clinically-related decisions, thereby mitigating discrepancies between the care they expect and what they receive (Manning et al., 2012).

## 3 Conclusion

Creating a positive context is essential for providing care that sets both patients and organizations up for success. But healthcare delivery is complex, with many actors and factors colliding within context. It is therefore crucial that we expand how we consider patient expectations and nocebo effects—thinking beyond of the patient-clinician interaction to the organization at large. Doing so could expose previously unknown, but significant, contributors to nocebo effects and transform the way organizations deliver care. The Society for Interdisciplinary Placebo Studies (SIPS) has stated that the knowledge and prevention of nocebo effects is a priority (Evers et al., 2018) and has promoted international educational projects for healthcare providers^1^ on this topic. We hope we have raised awareness by providing possible real-world organizational triggers of nocebo effects and implications for their management.

## Author contributions

DP: Writing—review & editing, Writing—original draft, Conceptualization. MM: Writing—review & editing, Writing—original draft, Conceptualization. JD: Writing—review & editing, Writing—original draft, Conceptualization. AP: Writing—review & editing, Writing—original draft, Supervision. GR: Writing—review & editing, Writing—original draft, Supervision, Conceptualization.

## Footnotes

^1^PANACEA Special Needs by Placebo: Programme to Advise, Normalize and Control its European Administration. (2022). Available online at: https://site.unibo.it/educationonplacebo/en.
